# Text message interventions for follow up of infants born to mothers positive for Chagas disease in Tucumán, Argentina: a feasibility study

**DOI:** 10.1186/s13104-015-1498-9

**Published:** 2015-09-29

**Authors:** Gabriela Cormick, Alvaro Ciganda, Maria L. Cafferata, Michael J. Ripple, Sergio Sosa-Estani, Pierre Buekens, José M. Belizán, Fernando Althabe

**Affiliations:** Department of Mother and Child Health Research, Institute for Clinical Effectiveness and Health Policy (IECS), Buenos Aires, Argentina; Unidad de Investigación Clínica y Epidemiológica Montevideo, Montevideo, Uruguay; Institute for Global Health and Infectious Diseases, University of North Carolina at Chapel Hill, Chapel Hill, NC USA; Instituto Nacional de Parasitología (INP), “Dr Mario Fatala Chaben”, Administración Nacional de Laboratorios e Institutos de Salud (ANLIS) Malbrán, Buenos Aires, Argentina; School of Public Health and Tropical Medicine, Tulane University, New Orleans, LA USA

**Keywords:** mHealth, Mobile health, Maternal health, Child health, Text message intervention

## Abstract

**Background:**

Diagnosis of congenital Chagas disease occurs at 9 months of age, making effective treatment challenging due to loss to follow-up. Mobile health (mHealth) has been utilized to improve communication and treatment adherence in many chronic diseases, although no studies of mHealth in *Trypanosoma cruzi*-infected individuals have been conducted. Text message interventions, a subset of mHealth, has shown to improve appointment attendance and is relatively simple to set up, thus making it an ideal mechanism to facilitate communication with individuals in low-resource settings.

**Objective:**

The aim of this study is to understand the acceptability, utilization, and barriers of an SMS-based appointment reminder to confirm a post-partum home visit to women in Tucumán, Argentina and whether these factors differ in urban and rural populations.

**Methods:**

Women that tested positive for Chagas disease were invited to receive SMS reminders of their follow-up 4-week postpartum home visit. Demographic information and SMS contact preferences were collected at hospital discharge, and variables on mHealth utilization and barriers were recorded at follow-up.

**Results:**

77 (70.6 %) of women possessed a cell phone for personal use. All eligible women owned phones compatible with SMS messages. The appointment reminder SMS was widely accepted with 64/72 (88.9 %) enrolled women receiving the SMS message and 58/64 (90.6 %) replying. Ninety-two percent of women stated that the text message was a useful reminder for the follow-up home visit. Women living in rural areas were less likely to own a cell phone for personal use and were significantly less likely to have internet access on their phone than women living in urban areas (RR 0.30, 95 % CI 0.10–0.89). Furthermore, women from rural areas faced barriers to mHealth uptake such as change of phone number and response to messages from the hospital team at higher rates than women from urban areas, although these differences were not statistically significant.

**Conclusions:**

There is generally widespread acceptance and utilization of mHealth among this group of women with access to cell phones. However, there are still many barriers to overcome before mHealth interventions attain complete penetration in a population, most notably the issue of cell phone for personal use.

## Background

Chagas disease, or American trypanosomiasis, is caused by the protozoan parasite *Trypanosoma cruzi*. It is a major cause of morbidity and mortality in the Americas due to the fact that pregnant women with Chagas disease can transmit *T. cruzi* to their fetuses [1–3]. The diagnosis and effective treatment of congenital infection can be performed in an infant after 9 months of age, following the disappearance of maternal antibodies. However, the time required for diagnosis often results in a loss of contact with the mother [4, 5]. It has been estimated that between 20 and 50 % of children born to infected women are diagnosed and treated appropriately [4, 6–8]. Contact with the families is crucial to improve diagnosis and a confirmed appointment with the family aids in preventing unnecessary house visits, thus reducing project costs.

Text message interventions (TMI) are an important part of the larger strategy of mobile health (mHealth), which entails the utilization of mobile technology in healthcare. Almost 85 % of residents of developing countries report cell phone ownership [9], making TMI an attractive tool to engage patients and improve communication with healthcare providers. Meta-analyses have demonstrated the largely positive effect of TMI on healthy behavior [10, 11], medication adherence [12], and smoking cessation [13]. The efficacy of text message reminders has been well established in existing literature [14–16]. However, of the 65 primary research articles referenced in these seven reviews, only eight studies were conducted in low- and middle-income countries (LMICs) and only one was conducted in Latin America [17]. Thus, while research on TMI is plentiful, the vast majority of it is conducted in high-income countries with more resources and infrastructure, or LMICs outside of Latin America, which have different socioeconomic and cultural influences.

Our previous research has shown the interest of pregnant women in Argentina to receive text messaging as a form of communication with healthcare staff [18]. Previous studies suggest that the acceptability and accessibility of a mHealth program may vary between urban and rural populations as individuals in rural areas are less likely to own a mobile phone and feel comfortable in their use [19, 20]. While mHealth interventions often recruit individuals from both urban and rural areas, little work has been done to determine similarities and differences in usage and access to mobile technology in these populations, in particular in Latin America.

The aim of this study is to understand the acceptability, utilization, and barriers of a SMS-based appointment reminder to confirm a post-partum home visit in Tucumán, Argentina. We also sought to elucidate whether there is a difference in these factors in urban and rural populations.

## Methods

This prospective observational study was carried out from December 2011 to April 2013 at the Instituto Maternidad Provincial Nuestra Señora de las Mercedes, a public maternity hospital located in the city of San Miguel de Tucumán, the capital city of Tucumán province. The maternity is the referral ward of northwest Argentina and has around 9000 deliveries per year. This study was nested within a study designed to detect the rate of congenital *T. cruzi* transmission in a cohort of seropositive women [21].

The inclusion criteria for the main study included all consecutive live births occurring from April 2011 to April 2013, irrespective of the gestational age or route of delivery (vaginal or cesarean). Only those women who tested positive for Chagas disease, had just delivered a live birth, were 18 years of age or older, and residing in the province of Tucumán area were invited to participate to the main study. The province has urban and rural areas. All women invited to the study were literate as they were all able to read and sign the informed consent.

The specific inclusion criteria for this nested study required participants to:

- be enrolled in the main study
- own a mobile phone for personal use,
- own a mobile phone compatible with SMS technology.

All eligible women were approached in person by data collection personnel and invited to participate in this study before being discharged from the hospital. Women were told that the purpose of the study was to evaluate the feasibility of using an SMS message as a reminder of their home visit, and if they accepted to participate they would be contacted by a text message to confirm the main study appointment at 4 weeks after delivery. They were also informed that they would receive credit to answer the text message. Those accepting to participate were asked to sign the informed consent in Spanish, which was approved by the Institutional Review Board of Tulane University and the Ethics Committee of the Center of Medical Education and Clinical Investigations in Buenos Aires, Argentina.

The study consisted of three phases, admission to the study before hospital discharge; visit reminder and a home visit at four-weeks after delivery. The admission form was administered face to face while women were still in the hospital. This form was used to evaluate participant´s date and time contact preferences and how many days in advance they would prefer to receive the reminder. Participants were also asked whether they lived in an urban or rural area. At this time, the hospital contact number was added to the participants phone address book to ensure the number was easily identified with the study and to avoid scams. The phone compatibility was checked sending a test text message from the study phone to the phone number provided by the woman during admission process.

According to the information on the admission form, the data collection team sent an SMS reminder to each participant in order to confirm the appointment for the 4-week home visit. Before sending the message the team transferred credit to each phone number so women could reply without having any extra cost. Information related to date and time of the reminder message as well as all replies from participants were transferred to a designed form in order to measure the outcome of interest.

Four weeks after delivery, the data collection team performed the scheduled home visits and completed another questionnaire in person to assess if the message had been received and the acceptability of the SMS appointment reminder. The questionnaire applied at 4-week follow up was a standardized questionnaire designed to record the reception of the message, the woman’s reply to the reminder message and her impression of whether the message was useful and whether the reception time was adequate. Reasons for not replying to the message and whether she had changed phone number was the same were also collected. A second part of the questionnaire assessed the general use of mobile phones such as time she had been using a mobile phone, frequency of changing the number, payment method, main use, internet access and if they used computers they were asked place of access, internet access, frequency use and main use. This second questionnaire had the aim of obtaining information to design future interventions. This questionnaire was adapted from our previous study [18]. The team also recorded their assessment of the urban or rural status of the participant’s house and this was compared to the participants’ responses at enrolment. These two sets of data were then compared to ensure consistency in stratifying patients as urban or rural.

The hospital team received training on interviewing techniques and information regarding mHealth and text messages. The completed questionnaires were stored at the hospital until collected by research staff.

### Data analysis

Data were entered into Open Clinica Version 3.1.1-Community and analyzed using SPSS version 21. The study population’s characteristics, access to cell phones and SMS message’s responses were reported as proportions. We used a Chi-squared test to assess the relationship between living in an urban or rural area and having a mobile phone for personal use. To study differences in access to and usage of mHealth between rural and urban populations we calculated the relative risk with a 95 % confidence interval. A *p* value of less than 0.05 was considered statistically significant.

## Results

### Enrollment and demographics

**Fig. 1:**
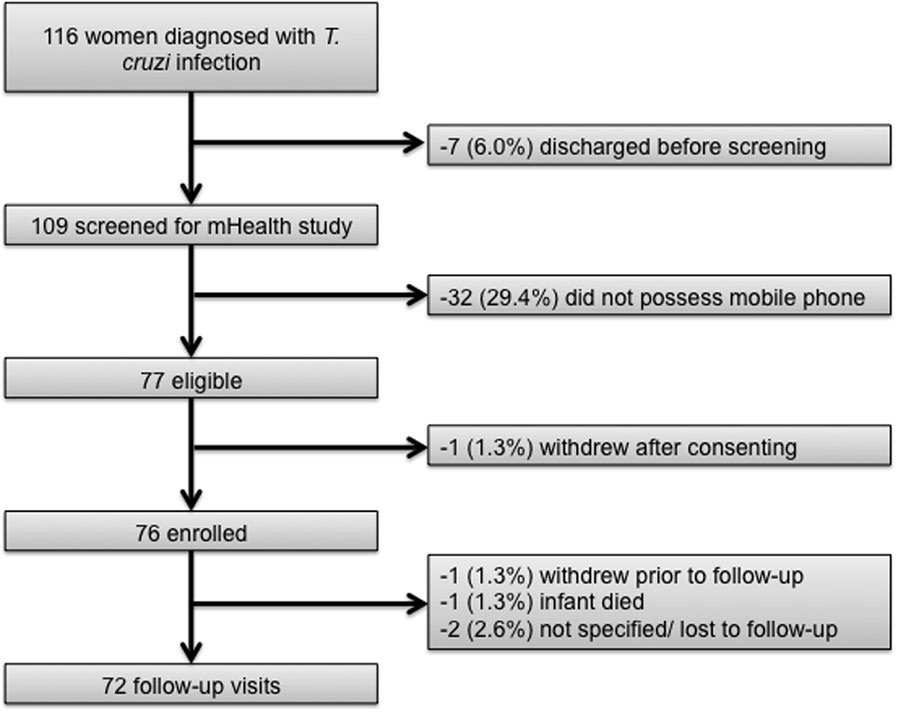
Flow chart of patient enrollment in the nested mHealth study

A total of 116 post-partum women meeting the inclusion criteria for the main Chagas study were identified during the recruitment period of the nested study and 109 were contacted during their postpartum stay and invited to participate in the nested mHealth study (Fig. 1). Of the 109 women screened for the study, 32 (29.4 %) women did not have a cell phone for personal use and were thus not eligible for the study. The remaining 77 (70.6 %) women were eligible and consented to participate, however one woman withdrew from the study after signing the informed consent. During the enrolment process the team stored the phone number of the maternity ward to ensure that the subjects would recognize the sender of the message in 66 of the subjects’ phone and they confirmed the reception of the test SMS in 70 (92.1 %) of cases.

Table 1 provides the demographic characteristics, SMS accessibility, and contact preferences of women interviewed. Six of the 76 participants (7.9 %) were 18–20 years old, and 12 participants (15.8 %) were older than 35 years. In addition, 36.8 % had also completed secondary school, and 2.6 % had finished a university education. Age and level of education of eligible women were similar to that of women in the main study. All participants were literate and had at least completed primary school. Forty-seven women (61.8 %) lived in urban areas, although participants were broadly distributed geographically in Tucumán Province, Argentina. Eligible women tended to live more in urban areas than non-eligible women.

**Table 1:** Demographic data and contact preferences (n = 76)

Characteristics	n (%)
Age	
Less than 20 years	6 (7.9)
20 to less than 35	58 (76.3)
35 or more	12 (15.8)
Area of residence	
Urban	47 (61.8)
Rural	25 (32.9)
Not available	4 (5.3)
Highest education level completed	
Less than primary education	0
Primary education completed	46 (60.5)
Secondary education completed	28 (36.8)
University degree completed	2 (2.6)
Signal in home	
Yes, always	73 (96.1)
Yes, sometimes	3 (3.9)
No, never	0
No answer	0
Cell phone can receive SMS	
Yes, sent message and verified	70 (92.1)
Yes, sent message but did not verified	5 (6.6)
Yes, but did not send message	1 (1.3)
No, called and verified	0
No, called but did not verify	0
Don’t know/no answer	0
How many days before the visit would you like to receive a reminder?	
1 Day	44 (57.9)
2 Days	26 (34.2)
3 Days	1 (1.3)
No answer	5 (6.6)
What time of day would you like to receive the message?	
Morning (8 am–12 pm)	18 (23.7)
Afternoon (12 pm–4 pm)	2 (2.6)
Evening (4 pm–8 pm)	8 (10.5)
Anytime	45 (59.2)
Don’t know/no answer	3 (3.9)

### Participant SMS compatibility and contact preferences evaluated at admission

All women enrolled in the study reported to have some level of cellular service in their home, with the vast majority 73 (96.1 %) stating that they always have service in their home and only three respondents stating that they sometimes have cell service at home (Table 1). Almost all participants preferred the reminder message to arrive 1–2 days before the follow-up visit. Most women did not have a preference with regard to the time of day to receive the reminder message.

### Follow-up reminder and home visit

The maternity team sent a text message reminder of the four-weeks home visit appointment to all 76 women, and 59 (76.6 %) replies were received (Fig. 1). Two women used this opportunity to cancel the home visit; one woman because the child had died and the other one asked to be withdrawn from the study and other two women were lost to follow-up.

The study team completed 72 four-week follow-up home visits. A total of 66 (91.7 %) women were using the same phone number registered at admission (Table 2). Out of the 72 women visited, 64 (88.9 %) reported having received the reminder message and 58 of those 64 (89.2 %) reported replying. Of the eight women that did not receive the reminder message, in five cases it was due to a change in their mobile number in the 4 weeks from enrollment to follow-up.

**Table 2:** mHealth usage and user satisfaction at follow-up

mHealth usage and user satisfaction at follow-up	Yes	n	%
The number being used at follow-up is the registered number	66	72	91.7
Did you receive the message from the monitoring group?	64	72	88.9
Did you respond to the message from the monitoring group?	58	64	89.2
Was the message a helpful reminder of the visit?	59	64	92.2
Was the time that you received the message appropriate?	57	64	89.1

Of the 64 women that received the message, 57 (89.1 %) reported that the reminder message arrived at a convenient time and an appropriate number of days in advance. Importantly, 59 (92.2 %) women stated that the message was useful, with 38 (59.4 %) women reporting that they would have forgotten about the appointment if not for the message.

### Usage of mobile technology among post-partum women

We also surveyed cell phone habits and practices of study subjects at the 4-week follow-up visit (Table 3). Length of cell phone ownership varied among the study group from less than 6 months to greater than 2 years. Likewise, age at first phone ownership varied from less than 15 years old to greater than 35 years old, with most women owning their first phone between 15 and 20 years old (38.9 %) or 20 and 35 years old (40.2 %). Of the 72 participants, 63 (87.5 %) had a prepaid cell phone plan, and only 13 (18.8 %) had the same phone number for more than 1 year.

**Table 3:** Questionnaire responses at follow-up (n = 72)

Characteristic	n (%)
Length of time having cell phone	
<6 months	24 (33.3)
6 months–<1 year	6 (8.3)
1 Year–<2 years	17 (23.6)
2 Years or more	20 (27.8)
Don’t know/no answer	5 (6.9)
Age at first cell phone	
Less than 15	5 (6.9)
15 To less than 20	28 (38.9)
20 To less than 35	29 (40.2)
35 Or more	1 (1.4)
No answer	9 (12.5)
Cell phone plan	
Contract	5 (6.9)
Prepaid	63 (87.5)
Don’t know/no answer	4 (5.6)
Changed cell number in the last 12 months	
Yes	45 (65.2)
No	13 (18.8)
Don’t know/no answer	14 (19.4)
Primary use of the phone	
SMS	20 (27.8)
Calls	3 (4.2)
Both	44 (61.1)
Don’t know/no answer	5 (6.9)
Use of internet on the phone	
Yes	24 (34.3)
No access	41 (58.6)
Don’t know/no answer	7 (9.7)
Regular access to the internet	
Yes	12 (17.4)
No	56 (81.2)
No answer	1 (1.4)

Upon questioning participants about the primary use of their phones, 20 (27.8 %) reported using them primarily for texting, while only 3 (4.2 %) reported using them primarily for making calls, and 44 (61.1 %) reported using their phone for both calls and texting. Additionally, we probed participants on their access to the internet and found that 24 (34.3 %) accessed the internet on their phones and 12 (17.4 %) reported having regular access to the internet on the computer (Table 3).

### mHealth in urban and rural populations

Subjects were split into urban and rural groups based on questionnaire data and confirmation at the home visit. Out of 109 individuals screened for the TMI study, 102 cases had complete data on urban and rural status. Within these 102 cases there were only two cases with discrepancies in urban or rural status between patient responses at enrollment and the data collected by the study team at follow-up. Upon review of the participant data, we found that in these two cases, the women had moved homes between enrollment and follow-up. In the group of 102 participants that were screened for the mHealth study, 75.8 % of individuals from urban areas possessed a mobile phone, compared to 62.5 % of rural individuals, although this difference was not statistically significant (p = 0.15).

There was a statistically significant difference in the use of internet on the subjects’ phones between urban and rural groups, with 50.0 % of urban participants reporting the use of internet on their phones compared to only 15.0 % of rural participants (RR 0.30, 95 % CI 0.10–0.89, p = 0.03). Other variables that were not statistically significant between urban and rural populations but that trended towards lower values in rural populations were the use of the same phone number at enrollment and follow-up (RR 0.87, 95 % CI 0.72–1.05; p = 0.16), change of phone number in the previous year (RR 0.62, 95 % CI 0.26–1.44; p = 0.26) and response to the message from the monitoring team (RR 0.87, 95 % CI 0.70–1.09; p = 0.24, Table 4).

**Table 4:** Markers of utilization of mHealth interventions in urban and rural populations

	Urban	Rural	RR rur/urb (95 % CI)	p
	n (%)	n (%)		
Phone at follow-up is the same as enrollment (n = 69)				
Yes	43 (95.6)	20 (83.3)	0.87 (0.72–1.05)	0.16
No	2 (4.4)	4 (16.7)		
Receive message from team (n = 69)				
Yes	41 (91.1)	20 (83.3)	0.91 (0.75–1.12)	0.22
No	4 (8.9)	4 (16.7)		
Respond to message from team (n = 62)				
Yes	38 (92.7)	17 (81.0)	0.87 (0.70–1.09)	0.24
No	3 (7.3)	4 (19.0)		
Number did not change in last 12 months (n = 65)				
Yes	17 (38.6)	5 (23.8)	0.62 (0.26–1.44)	0.26
No	27 (61.4)	16 (76.2)		
Use of internet on phone (n = 62)				
Yes	21 (50.0)	3 (15.0)	0.30 (0.10–0.89)	*0.03*
No	21 (50.0)	17 (85.0)		
Type of cell phone plan (n = 65)				
Contract	3 (6.8)	1 (4.8)	0.70 (0.077–6.32)	0.75
Prepaid	41 (93.2)	20 (95.2)		

Italic indicates statistically significant p value

## Discussion

In this study we assessed the feasibility of using a SMS reminder in a group of post-partum women in Argentina who tested positive for *T. cruzi* infection. We found that approximately 70 % of these women possessed a cell phone for personal use and were eligible for the study. All eligible women in the study owned phones that were compatible with SMS messages and had a cell phone signal in their home at least some of the time. The appointment reminder SMS reached approximately 90 % of those individuals that participated in this study. Importantly, the SMS reminder was widely accepted and considered a useful reminder of the follow-up home visit that is crucial for the successful treatment of their children. The two primary barriers to the utilization of this program were the ownership of a mobile phone that prevented women from enrolling this study and a changed mobile phone number that compromised their follow up. Although this study did not have the statistical power to detect a difference, in general, the prevalence of barriers to mHealth utilization was higher in rural than urban populations.

Most women had the same preference to receive messages 1 or 2 days before the appointment and most had no preference on the reception time making feasible to scale up this intervention.

Although most women had a cell phone for personal use, the percentage was lower than the near universal use that we had previously assessed in pregnant women in other areas of Argentina [18]. Additionally, a recent survey of cell phone usage in developing nations found that approximately 83 % of Argentineans own a cell phone [9], similarly higher than the cell phone ownership rate we found in this study. These discrepancies may be due to number of women with shared phones and not exclusively for personal use, or due to regional, social, and/or economic factors in Tucumán province. Regardless of the cause of the discrepancy, these data indicate that while 70–80 % of the population would be eligible for an SMS-based reminder system, there remain a substantial number of individuals who do not own a phone and therefore do not have access to SMS-based resources and this limitation is higher for those who live in rural areas.

In addition, changes of phone number are a common occurrence and would pose a challenge with any long-term mHealth study relying on the use of the patients’ phones due to the fact that approximately 8 % of participants changed their phone number in only 4 weeks between enrollment and follow-up. Moreover, 65 % of participants reported changing their number in the previous 12 months. Thus, changes in participant phone number represent a significant barrier to the effective implementation of an SMS reminder program in studies with a prolonged follow-up period. We did not investigate the causes of the frequent changes of phone number, but one potential reason would be the frequency offers of low cost cellular service for new accounts which influences individuals to change their service provider and phone number.

There is also a trend of increased barriers to the access to or utilization of mHealth in rural populations; compared to women from urban areas, women from rural areas were less likely to possess a cell phone, less likely to respond to messages from our monitoring team, and more likely to have changed their phone number, either between enrollment and follow-up or as self-reported in the previous 12 months. These data suggest that while urban and rural groups face similar barriers in their access to mHealth, rural populations encounter these barriers more frequently. This is particularly important because one of the often-stated benefits of mHealth is the potential to extend health care and health information to geographically distant or isolated locations [22–24]. Challenges facing the implementation of a TMI may be significantly more common in rural populations and must be adequately addressed to ensure success of the intervention.

The strengths of this study include the in-depth questionnaires at enrollment and follow-up to assess feasibility and acceptability of a 2-way text message system. Additionally, subject location data allowed us to compare barriers to mHealth utilization in urban and rural populations. These findings can be generalized to Chagas positive population in Argentina as the population who participated in this study is comparable to the population attended in public hospitals in Argentina. However, because this was a study nested in a main study on congenital transmission of *T. cruzi*, we only enrolled post-partum women with confirmed *T. cruzi* infection. This limits the replicability of the study for its general use in other population. Fewer women owned a telephone and more women lived in rural areas when comparing this Chagas positive population with our previous study of non-positive population [18]. As a result, caution should be exercised in extending the results of this study to the general population.

Implementation of any mHealth project faces certain challenges regardless of the setting. These include adjusting to evolving technology, the selection of a mobile phone and platform that will be utilized and accepted by the study population, and the specifics of the study population, i.e. poor eyesight, impaired motor skills, etc. Implementing an mHealth study in a rural area and/or resource-limited setting necessitates the adaptation to these and other challenges, such as the availability and stability of a cellular network and electrical grid. Additional considerations include the availability of personal cellular devices, privacy and security of information, sufficient mobile credit to make calls and send/receive messages, and the frequency with which users change phone numbers. Our study has begun to elucidate the prevalence of these barriers in urban and rural populations in Tucumán, Argentina. Future studies will consider these barriers when designing and implementing an effective mHealth intervention utilizing SMS communication or a mobile internet connection.

## Conclusions

There was a widespread acceptance and utilization of mHealth among this group of women with access to cell phones. However, there are still many barriers to overcome before mHealth interventions attain complete penetration in a population, most notably the issue of individual cell phone ownership. Future studies should investigate the efficacy of text message reminders on follow-up visits for infants born to mothers infected with *T. cruzi* to test for congenital transmission, and also when necessary, its efficacy as reminder of appropriate treatment.
